# Chikungunya: An Emerging Public Health Concern

**DOI:** 10.1007/s11908-022-00789-y

**Published:** 2022-11-17

**Authors:** Omar Mourad, Leila Makhani, Lin H. Chen

**Affiliations:** 1 Division of Infectious Diseases, McMaster University, Hamilton Health Sciences, Hamilton, ON Canada; 2 Department of Family and Community Medicine, University of Toronto, and Tropical Disease Unit, Toronto General Hospital, Toronto, ON Canada; 3 Division of Infectious Diseases and Travel Medicine, Mount Auburn Hospital, Cambridge, MA USA; 4 Harvard Medical School, Boston, MA USA

**Keywords:** Alphavirus, Arbovirus, Arthritis, Infectious diseases, Travel, Vector-borne disease, Importation

## Abstract

**Purpose of Review:**

The worldwide spread of chikungunya over the past two decades calls for greater knowledge and awareness of the virus, its route of transmission, methods of diagnosis, and the use of available treatment and prevention measures.

**Recent Findings:**

Chikungunya virus infection, an *Aedes* mosquito-borne febrile disease, has spread from Africa and Asia to Europe and the Americas and from the tropics and subtropics to temperate regions. International travel is a pivotal influence in the emergence of chikungunya as a global public health threat, as evidenced by a growing number of published reports on travel-related chikungunya infections. The striking features of chikungunya are arthralgia and arthritis, and the disease is often mistaken for dengue. Although mortality is low, morbidity can be profound and persistent. Current treatment for chikungunya is supportive; chikungunya vaccines and therapeutics are in development. Travelers planning to visit areas where the mosquito vectors are present should be advised on preventive measures.

**Summary:**

Chikungunya is an emerging disease in the Americas. Frequent travel, the presence of at least two competent mosquito species, and a largely naïve human population in the Western Hemisphere create a setting conducive to future outbreaks. Awareness of the disease and its manifestations is critical to effectively and safely manage and limit its impact. Vaccines in late-stage clinical trials offer a new pathway to prevention.

## Introduction

The COVID-19 pandemic has dominated the world of infectious diseases, and the world itself, since March 2020. As of October 2022, COVID-19 has claimed more than 6.5 million lives globally [1], including more than 1 million in the USA, 687,000 in Brazil, 528,000 in India, and 2 million throughout Europe [1, 2]. The rapid spread of the SARS-CoV-2 virus and its variants dramatically demonstrates the capacity of infectious diseases to move around the world with relative ease in an era of abundant international travel and commerce. It is not a new phenomenon. Travel has long played an influential role in the global spread of infectious disease, from measles, influenza, and tuberculosis to, more recently, severe acute respiratory syndrome (SARS), Zika, and now COVID-19.

An emerging disease on this list is chikungunya, a mosquito-borne viral infection that causes debilitating joint and muscle pain. The acute symptoms typically resolve within a week to 10 days, but in some cases arthritis and arthralgia persist for weeks, months, or even years in a condition mimicking rheumatoid arthritis [3–6, 7•]. The impact on productivity, quality of life, and physical and mental well-being can be substantial. No antiviral treatment or vaccine is currently available although drugs and vaccines are in development [8•].

With the exception of mother-to-child transmission, chikungunya is not spread person-to-person [9, 10•, 11•]. Mosquitoes become infected by feeding on humans or non-human primates with a high level of virus in their bloodstream. Infected mosquitoes can then infect other humans. In areas of the world where the chikungunya virus is circulating, sudden outbreaks with high attack rates can affect one-third to three-fourths of the population [12, 13]. In Europe and North America, reported cases of chikungunya have largely been limited to travelers returning from parts of the world where the disease is epidemic or endemic. Epidemiologists, infectious disease specialists, and the travel medicine community are concerned, however, that the elements enabling outbreaks are in place: frequent travel from one part of the world to another, two competent mosquito vectors of chikungunya with an established presence in the Western hemisphere, and a largely naïve human population [14•].

This article will review the history and epidemiology of chikungunya, the clinical features of acute and chronic disease, the use of diagnostic tests, the available treatment options, and preventive measures. The pre-travel consultation for persons planning international travel is important in providing advice to reduce risk for the disease and to discuss the symptoms in returning travelers that raise concern for chikungunya.

## A Newly Global Phenomenon

**Fig. 1:**
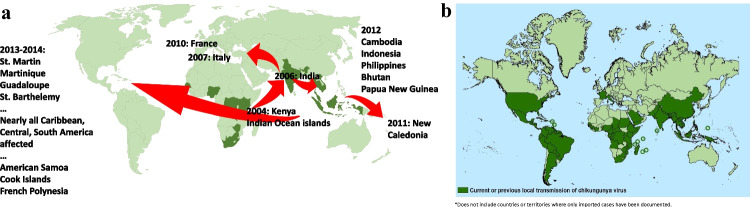
Countries and territories where local transmission of chikungunya in humans has occurred. **a** Dark green areas indicate reported local transmission as of 2004, when an outbreak in Lamu Island, Kenya subsequently spread to different regions (sources: data from Bettis et al. [18••], Weaver and Lecuit [19••], and Weaver [20]). **b** Dark green areas show ongoing or previous local transmission of chikungunya as of March 2022, demonstrating spread to the Americas over the past decade (source: CDC [21])

First recognized in what is now Tanzania in 1952 [15, 16], chikungunya has been reported from every continent except Antarctica [17•] and has spread over the past two decades from Asia and Africa to Europe and the Americas and from the tropics and subtropics to temperate regions (see Fig. 1a, b). A literature review of the epidemiology of chikungunya from 1999 to 2020, encompassing 97 outbreak reports from 45 countries, confirms the disease’s status as an emerging global public health concern [18••].

Chikungunya virus caused sporadic outbreaks in Africa and Asia for decades but did not vault to public attention until an outbreak in coastal Kenya in 2004 spread to Indian Ocean islands and then to India, where it caused explosive epidemics [19••], starting the “global onslaught” [22•]. By one estimate, the epidemic reached more than 100 countries and resulted in more than 10 million cases [6]. Cases imported by travelers found their way into Europe and local transmission followed, with 205 cases reported in northern Italy in 2007 [23]. The index case was a traveler from India who was visiting a relative in Italy. The 2007 outbreak was the first reported autochthonous transmission in a temperate region due to the wide distribution of the vector, *Aedes albopictus*, in many parts of Europe and the USA [24•]. The presence of *Aedes albopictus* in Italy had been documented in the early 1990s, in a warehouse of a tire retreading company, where used tires imported from the southeastern USA were infested with mosquito eggs. A second outbreak of locally transmitted chikungunya occurred in Italy in 2017 with approximately 400 total cases [25•]. While more than half the cases spread within the immediate area of the outbreak, secondary cases were documented as far as 60 miles away [26].

In late 2013, the first documented cases of locally transmitted chikungunya in the Western Hemisphere occurred in the Caribbean island of St. Martin. The disease then spread rapidly throughout the Caribbean and Central and South America [14•, 27]. As of late 2020, outbreaks of chikungunya in the Americas had resulted in an estimated 3 million cases [28]. A number of travel-associated chikungunya cases and outbreaks have been reported in association with the expansion since 2004, including from the GeoSentinel Surveillance Network [29, 30••, 31, 32•, 33–42, 43•, 44••, 45••, 46, 47] (see Table 1).

**Table 1:** Travel-associated chikungunya in the literature

**Years of occurrence**	**Number of traveler cases**	**Travelers’ country of residence**	**Countries or regions where exposure occurred**	**Comments**	**Reference**
1997–2010	40	GeoSentinel sites worldwide	Indian Ocean Islands	Most cases correlate with a regional outbreak of chikungunya in 2005–2006	Savini et al. [34]
1998–2018	744	Primarily Europe	Various	20-year analysis of EuroTravNet surveillance data; 608 of the 744 cases (82%) were reported from 2013 to 2018	Grobusch et al. [35]
2001–2017	113	New Zealand	Primarily Pacific Islands and Southeast Asia	Cases coincided with rainy seasons in destination countries	Ammar et al. [36]
2005–2006	12	USA	India, eastern Africa, Réunion Island	CDC’s first published report on chikungunya acquired by US travelers	CDC [37]
2006	26	USA	India, Sri Lanka	86% of cases in 2005–2006 involved travel in India	CDC [38]
2003–2015	75	France	Caribbean, Sub-Saharan Africa, Asia	Chikungunya cases increased and malaria and influenza-like illness decreased in the 12-year period studied	Griffiths et al. [39]
2006–2016	89	Japan	Asia (Indonesia, Philippines, India), Pacific and Caribbean islands, Latin America	Authors noted the “huge 2-way human traffic between Japan and chikungunya fever-endemic regions”	Nakayama et al. [40]
2007–2019	198	Taiwan	Primarily Myanmar; travel alerts also issued for Thailand, India, Maldives	> 40% of reported imported cases occurred in 1 year (2019)	Chou et al. [41]
2009–2018	280	Spain	Sub-Saharan Africa, Central America and Caribbean, South America, South Central Asia, Southeast Asia, Australasia, Europe	More than half of cases (160 of 280) were reported in people returning to Spain after visiting friends and relatives in their country of birth	Norman et al. [42]
2013–2017	> 4000	USA	Worldwide	12 locally acquired cases were also identified in the USA	Adams et al. [43•] Lindsey et al. [44••] Staples et al. [45••]
2013–2016	15	Europe	Brazil	Part of a risk analysis done prior to the 2016 Olympics in Rio de Janeiro	Gautret et al. [46]
2019	5 of 11 relatives traveling together	Finland	Thailand	1 child in ICU, 3 adults hospitalized, 3 with persistent arthralgia	Kantele [47]
2019	9	Sweden, Switzerland, UK, Romania, Israel, France	Thailand	Time spent abroad ranged from 10 to 35 days, 4 patients hospitalized	Javelle et al. [48]
2019	18	France, Germany, Italy, Japan, Laos, Spain, USA	Myanmar	14 patients experienced arthralgia	Diaz-Menéndez et al. [49]
2019–2020	8	Denmark, Germany, Spain, France, Italy	Maldives	Mean duration of stay was 11 days; 2 patients hospitalized	Dudouet et al. [50]

Travel and international commerce are substantial contributing factors to the global spread of chikungunya disease [13, 27–29, 30••, 31, 32•, 33–42, 43•, 44••, 45••, 46–48]

Chikungunya among US travelers is a recent phenomenon [33, 37, 38, 43•, 44••, 51•]. From 2006 to 2013, annual cases ranged from 5 to 65 [44••]. In 2014, 2799 cases of chikungunya among US travelers were reported to ArboNET, a national arboviral surveillance system of the Centers for Disease Control and Prevention (CDC) in collaboration with state health departments [51•]. In addition, 12 locally acquired cases were identified in the continental US, the first report coming from Florida [52]. Chikungunya also arrived in US territories, mainly Puerto Rico [53] and the US Virgin Islands [54]. More than 4600 locally acquired infections were reported in US territories in 2014. Since that time, small numbers of locally transmitted cases have been reported annually in the territories [51•].

Chikungunya became a nationally notifiable disease in the USA in 2015. In that year, 896 cases were reported, all but one related to travel, with the highest number reported from California, New York, Florida, and Texas, where one case of locally transmitted disease occurred [51•]. In subsequent years, reported cases have ranged from a high of 248 in 2016 to a low of 33 in 2020, a year in which the COVID-19 pandemic severely restricted international travel. All reported cases from 2016 through 2020 occurred in travelers except for one case attributed to laboratory transmission [51•].

The European Centre for Disease Prevention and Control (ECDC) collects data on chikungunya infections reported by countries across the European Union and European Economic Area [55]. In 2014, the agency reported 875 confirmed cases, due in large measure to travel-related importation from the Caribbean and the Americas. Since then, annual confirmed cases in Europe have ranged from 478 (2015) and 461 (2017) to 113 (2018) and 52 (2020). The vast majority of these are imported cases; locally acquired cases were reported sporadically in Italy and France in 2007, 2010, 2014, and 2017 [55].

Clinicians should consider chikungunya in travelers who present with fever, rash, and arthralgia or arthritis and are returning from areas of the world with known chikungunya transmission, including the Caribbean [46, 56]. In addition, chikungunya should be considered in the differential of febrile patients with exposure in areas with competent mosquitos. For instance, recreational history is relevant. In 2020, a 31-year-old woman in Florida with fever, headaches, and mild swelling of the hands was treated empirically, first for meningitis and then for Lyme disease, before being diagnosed with chikungunya infection, most likely acquired locally during a hiking trip [57].

## The Virus and Vectors

Chikungunya is an arthropod-borne virus (arbovirus) along with Zika, dengue, and West Nile. The virus exists as a single serotype [58] with three different lineages or genotypes identified: East Central Southern African (ECSA), West African, and Asian. A fourth, Indian Ocean, is considered an offshoot of the ECSA lineage.

The chikungunya virus is transmitted primarily by the female *Aedes aegypti* and *Aedes albopictus* mosquitoes, the same mosquitoes that transmit dengue and Zika virus. They typically bite, aggressively, during the daytime. *Aedes aegypti* has been described as a “nervous feeder”; if a blood meal is interrupted, it will move on to another person, producing clusters of infection within a household [22•]. Epidemics tend to occur during the rainy seasons in tropical areas.

Blood-borne transmission has been documented and in utero or intrapartum transmission is possible but rare. The highest risk for maternal–fetal transmission occurs when an infected mother is highly viremic around the time of delivery [45••]. Otherwise, person-to-person transmission is not known to occur. While there have been no documented cases of transmission via organ transplantation, the virus has been detected in corneal grafts suggesting that this may be a possibility [59]. Chikungunya virus has been detected during testing of donated blood products, the CDC notes, although no transfusion-related cases have yet been identified [45••]. Strategies to minimize the possibility of transfusion-related transmission of chikungunya include enhanced pre-donation symptom screening, post-donation illness notification, and deferral of donations from at-risk individuals [60].

Humans serve as the primary host of the virus during epidemics. Anyone with suspected chikungunya infection should avoid mosquito exposure for at least 7 days after the onset of illness to reduce the possibility of transmitting the virus to mosquitoes, which could then transmit to other humans. The complete transmission cycle from human to mosquito and back to another human can take place in less than a week [13]. Once a mosquito is infectious, it may be capable of transmitting the virus for the remainder of its lifespan (about 2 weeks).Infection with chikungunya virus confers long-lasting, possibly lifelong, immunity [13, 61].

## Disease Manifestations and Persistence

Symptomatic disease develops in the majority of infected individuals, usually 2 to 6 days after the mosquito bite, with a range of 1 to 12 days [19••, 45••, 62]. Abrupt onset of high fever (> 102 °F) is accompanied by severe joint and muscle pain. “Chikungunya” comes from a word in the Kimakonde language of Tanzania and Mozambique meaning “to become contorted,” a reference to the stooped appearance of people suffering intense arthralgias [19••]. The intensity of chikungunya symptoms corresponds to the level of viremia, which is highest during the first few days of infection. Joint pain is bilateral and symmetric, most often involving the wrist and hands and the ankles and feet but may affect knees and elbows and other joints as well. Morning stiffness may be severe. Other acute symptoms include headache, back pain, fatigue, nausea, joint swelling, conjunctivitis, and, in about half of affected individuals, an erythematous maculopapular rash. The rash may be mild and localized or extensive, encompassing more than 90% of the skin [4].

The differential diagnosis of chikungunya includes many viral infections, particularly dengue, Zika, and other alphaviruses depending on geographic exposure (e.g., Mayaro, Ross River virus, Barmah Forest virus, o’nyong’nyong, and Sindbis virus) [45••]. Given appropriate travel exposures, diagnoses may include leptospirosis and malaria. Globally distributed infections such as adenovirus, enterovirus, measles, parvovirus, and rubella may also manifest in similar fashion [45••]. Also, non-infectious inflammatory arthritis should be considered in the differential.

Cardiac manifestations are of particular importance due to the high associated mortality [63–65, 66••]. In a 2005–2006 outbreak on Réunion Island, a French department in the Indian Ocean, 610 cases (0.3% of all cases) were characterized as having atypical presentations [64]. Among the atypical cases, mortality was 10.6%; heart failure accounted for 23% of deaths, myocarditis and pericarditis 8%, and myocardial infarction 3%. A majority of patients who developed these manifestations had a previous history of cardiovascular disease [64, 67•]. Postmortem biopsy in one patient who developed fulminant myocarditis (with no remarkable medical history) showed necrotic lesions and cytoplasmic viral inclusions in the cardiac tissue [65].

The most common neurologic complication of chikungunya is encephalitis, occurring in the Réunion Island outbreak at a rate of 8.6 per 100,000 persons [68•]. Classically, encephalitis develops in the acute phase of infection, often within the first 24 h following the onset of high fever [68•, 69–71]. Cases of seizures, meningoencephalitis, and Guillain–Barre syndrome have also been reported, although at less frequent rates. A review of neurologic complications in 130 cases reported a range of outcomes, from direct central nervous system infections in infants and the elderly with comorbid conditions to autoimmune-mediated complications in previously healthy adults [70]. Neonatal infection has also been reported to be associated with neurologic involvement and cognitive impairment [71, 72], although a recent retrospective observational study of 150 mother–infant pairs infected with chikungunya during pregnancy did not identify increased pregnancy complications or adverse neonatal outcomes [73•].

Most symptomatic chikungunya infections resolve within a week to 10 days, followed by a post-acute stage that may last for 3 months after the onset of illness. Beyond that time, up to 40% of patients may experience painful, incapacitating symptoms that persist for months or even years in a post-chikungunya chronic polyarthralgia or arthritis, often accompanied by chronic fatigue and depression [5, 7•, 74, 75, 76•]. Long-term disability carries economic and psychosocial consequences for individuals who are unable to work [17•]. Our knowledge about post-chikungunya syndrome may help inform our understanding of “long COVID” and vice versa [77].

A systematic review of 34 studies found that 43% of cases did not fully resolve within 3 months after onset [78]. Following the outbreak on Réunion Island, 57% of patients continued to experience rheumatic manifestations 15 months after the acute illness [79]. Variables associated with persistence included age of 45 years or older, severe acute pain in the initial episode, and comorbid osteoarthritis. In a review of 65 cases in Colombia, more than half of patients had at least one rheumatologic symptom that persisted for more than a year after infection, and 43% reported post-chikungunya chronic polyarthralgia [75]. While the overall case fatality rate is estimated to be 0.13–0.14% [80•], mortality is significantly higher (> 10%) among patients who have comorbidities such as diabetes or develop cardiac or neurologic involvement, or possibly those co-infected with dengue or Zika [64, 66••, 81, 82•].

## Diagnostic Challenges

Preliminary diagnosis relies on the individual’s clinical presentation and a thorough travel history. Chikungunya can be misdiagnosed as dengue and even Zika in areas where the viruses are circulating, as their clinical features overlap. Co-infections can occur [17•, 83•, 84–89] (see Table 2).

**Table 2:** Clinical differences between dengue, chikungunya, and Zika virus infection [4, 62, 82•, 90••]

	**Chikungunya**	**Dengue**	**Zika**
**Incubation period (days)**	1–12	3–14	1–12
**Estimated proportion of infections that are asymptomatic (%)**	25	75	50–80
**Clinical**			
** Fever**	+++	+++	+
** Headache**	+	+++	+
** Retro-orbital pain**	+	+++	+
** Rash**	+	+++	+++
** Conjunctivitis**	+	++	+++
** Arthralgia/arthritis**	+++	+	+
** Myalgia**	++	+++	++
** Petechiae**	+	+++	+

+++ Indicates most likely/most frequent association

Confirming the diagnosis requires laboratory testing. The more widely available tests include polymerase chain reaction (PCR) to detect viral RNA in the first 8 days of illness, or acute-phase serology to detect IgM, IgG, and neutralizing antibodies toward the end of the first week of illness (> 4 days post-onset) paired with a convalescent-phase serology; although possible, viral cultures in the first 3 days of illness are less frequently used [91•, 92]. Confirmatory testing for neutralizing antibodies is available from reference laboratories such as the CDC’s Division of Vector-Borne Diseases (https://www.cdc.gov/ncezid/dvbd/index.html). Serological cross-reactivity can occur with other alphavirus infections, including Mayaro, o’nyong’nyong, Semliki Forest virus, eastern equine encephalitis, western equine encephalitis, and Venezuelan equine encephalitis. Investigators have also detected chikungunya virus in saliva, urine, and other bodily fluids in both the acute and convalescent phases [93, 94].

## Key Steps in Treatment and Prevention

Immediate treatment of chikungunya infection is supportive and symptomatic, emphasizing rest, fluids, and relief of pain. Patients with suspected chikungunya should be managed as though they had dengue (e.g., with acetaminophen or paracetamol rather than aspirin or nonsteroidal anti-inflammatory drugs) until dengue has been ruled out, due to the increased risk of bleeding and potentially life-threatening hemorrhage associated with the use of aspirin and other NSAIDS. Once a diagnosis of chikungunya without dengue co-infection is confirmed, NSAIDS, corticosteroids, and/or physiotherapy may help relieve persistent joint pain. Treatment of chronic chikungunya arthritis will likely require rheumatology, physiotherapy, and pain management expertise [7•].

A Latin American Consensus Conference held in 2017 issued a number of recommendations for the diagnosis and treatment of chikungunya-related inflammatory arthropathies [95•]. In the chronic phase, when polyarthritis persists for more than 3 months along with elevated erythrocyte sedimentation rate and C-reactive protein levels, and the patient does not respond to recommended doses of NSAIDs or steroids, the group suggests the use of disease-modifying antirheumatic drugs (DMARDs). The first choice is methotrexate (7.5 to 25 mg/week) or sulfasalazine (1–3 g/day); either could be used in combination with antimalarials such as chloroquine or hydroxychloroquine. If DMARDs fail, biological therapy with an anti-TNF-α agent is the next recommended option [95•]. For travelers, high-risk areas for chikungunya are destinations where competent mosquitoes are endemic, including the Americas, much of Africa, and Southeast Asia. Persons planning to visit any of these areas should seek pre-travel consultation. Although COVID-19 has disrupted international travel, the trend of increasing travel to tropical and sub-tropical areas warrants awareness of chikungunya and its preventive measures. In the absence of specific treatment, the individual preventive strategies include vector avoidance through the use of insect repellent, wearing long sleeves and long pants, treating clothing and gear with 0.5% permethrin, and staying in well-screened accommodations (alternatively, use a mosquito net) [45••]. Control measures in the community include eliminating standing water from places indoors and outdoors where mosquitoes can lay their eggs, such as buckets, planters, birdbaths, and trash bins. These strategies are applicable to long-term travelers and expatriates as well as residents in endemic areas. Public health measures to control mosquito populations are an important element of chikungunya prevention and focus on reducing the artificial and natural water habitats and containers that make breeding possible.

Therapeutics and vaccines for chikungunya are in active development. Vaccine candidates include the following:

- A single-dose live attenuated vaccine candidate that has completed a phase III immunogenicity and safety trial in the USA [96•], with results, reported in March 2022: 98.9% of participants achieved protective levels of neutralizing antibodies 1 month after a single vaccination and 96.3% achieved such levels 6 months post-vaccination [97].
- A virus-like particle vaccine candidate; a phase II study employing one- and two-dose schedules showed a “robust and durable” serum neutralizing antibody response [98]. Phase III trials evaluating the safety and immunogenicity of a single injection are under way with completion expected in November 2022 and March 2023 [99•, 100].
- A live recombinant measles-vectored vaccine candidate, which demonstrated “excellent safety and tolerability and good immunogenicity” in a phase II trial [101•, 102].

Drugs being evaluated for use in treating chikungunya infection include antivirals currently licensed in the USA or elsewhere for other purposes, such as ribavirin, arbidol, suramin, favipiravir, and sofosbuvir [8•, 17•, 103•, 104–106]. Other ongoing research on therapeutics ranges from IFN-α and chloroquine to imipramine and ivermectin, along with a large number of investigational agents in early stages of development [8•, 17•, 103•, 104, 107–109].

## Conclusion

Chikungunya virus infection has emerged as a global health threat over the past two decades, expanding to all continents except Antarctica. The disease has low mortality but high morbidity, including the risk of lifelong disability and its economic and psychosocial consequences. Widespread international travel and the presence of two competent mosquito vectors in most parts of the world create a setting for future outbreaks. Travelers heading to destinations where mosquitoes carrying the virus are endemic should seek pre-travel consultation. The emergence of chikungunya as a global public health concern calls for greater knowledge and awareness of the virus, its route of transmission, methods of diagnosis, and the use of available treatment and prevention measures [110••] (see Table 3).

**Table 3:** Selected chikungunya resources for practitioners and patients

**Resource**	**URL**	**Comments**
ArboNET	https://www.cdc.gov/mosquitoes/mosquito-control/professionals/ArboNET.html	National arboviral surveillance system of the CDC in partnership with state health departments
CDC Yellow Book 2020. Health Information for International Travel	https://wwwnc.cdc.gov/travel/yellowbook/2020/travel-related-infectious-diseases/chikungunya	Published every 2 years (with ongoing online updates) as a resource for health professionals caring for international travelers. Latest travel health guidelines, including pretravel recommendations and destination-specific advice
CDC website	https://www.cdc.gov/chikungunya/index.html	Information for the public as well as data and resources for healthcare professionals
CDC Division of Vector-Borne Diseases	https://www.cdc.gov/ncezid/dvbd/index.html 970–221-6400	Information on bacteria and viruses spread by mosquitoes, ticks, fleas, and other vectors. Specimens can be submitted to the division’s diagnostic laboratories
ECDC website	https://www.ecdc.europa.eu/en/chikungunya-virus-disease	Factsheets, infographics, communications toolkit, annual epidemiological summaries, and weekly threat reports, among other resources for European practitioners
International Society of Travel Medicine	https://www.istm.org	ISTM’s Travel Medicine Review and Update Course (https://learning.istm.org/tmruc) includes a module on vector-borne diseases
GeoSentinel	https://www.istm.org/geosentinel	Global surveillance network of the ISTM in partnership with CDC. Its 60 affiliated travel medicine clinics “are ideally situated to effectively detect geographic and temporal trends in morbidity among travelers, immigrants and refugees”
American Society of Tropical Medicine and Hygiene	https://www.astmh.org	The Society offers an examination (CTropMed) leading to a Certificate of Knowledge in Tropical Medicine and Travelers’ Health
World Health Organization	https://www.who.int/health-topics/chikungunya#tab=tab_1	Fact sheets, clinical guidelines, and reports on disease outbreaks around the world
Pan American Health Organization (PAHO)	https://www.paho.org/en/topics/chikungunya	Maps, data, fact sheets, and historical perspective on chikungunya disease in the Americas
PAHO’s Health Information Platform for the Americas (PLISA)	https://www3.paho.org/data/index.php/en/mnu-topics/chikv-en.html	An excellent platform from PAHO for more detailed epidemiology

## References

[1] Johns Hopkins Coronavirus Resource Center. COVID-19 Dashboard by the Center for System Science & Engineering at Johns Hopkins University. 2022. https://coronavirus.jhu.edu/map.html. Accessed 26 Oct 2022.

[2] Centers for Disease Control and Prevention (CDC). COVID Data Tracker. 2022. https://covid.cdc.gov/covid-data-tracker/#datatracker-home. Accessed 26 Oct 2022.

[3] Amaral JK, Bitsborrow JB, Schoen RT (2020). Chronic chikungunya arthritis and rheumatoid arthritis: what they have in common. Am J Med.

[4] Burt FJ, Chen W, Miner JJ (2017). Chikungunya virus: an update on the biology and pathogenesis of this emerging pathogen. Lancet Infect Dis.

[5] Sharma SK, Sanjay J (2018). Chikungunya: a rheumatologist’s perspective. Int J Rheum Dis.

[6] Suhrbier A (2019). Rheumatic manifestations of chikungunya: emerging concepts and interventions. Nat Rev Rheumatol.

[7] Zaid A, Gérardin P, Taylor A (2018). Chikungunya arthritis: implications of acute and chronic inflammation mechanisms on disease management. Arthritis Rheumatol.

[8] Powers AM (2017). Vaccine and therapeutic options to control chikungunya virus. Clin Microbiol Rev.

[9] Contopoulos-Ioannidis D, Newman-Lindsay S, Chow C, LaBeaud AD (2018). Mother-to-child transmission of Chikungunya virus: a systematic review and meta-analysis. PLoS Negl Trop Dis.

[10] de Ferreira FCPADM, da Silva ASV, Recht J (2021). Vertical transmission of chikungunya virus: a systematic review. PLoS One.

[11] Torres JR, Falleiros-Arlant DH, Dueñas L (2016). Congenital and perinatal complications of chikungunya fever: a Latin American experience. Int J Infect Dis.

[12] Gerardin P, Guernier V, Perrau J (2008). Estimating Chikungunya prevalence in La Réunion Island outbreak by serosurveys: two methods for two critical times of the epidemic. BMC Infect Dis.

[13] World Health Organization (WHO). Chikungunya. 2022. https://www.who.int/news-room/fact-sheets/detail/chikungunya. Accessed 26 Oct 2022.

[14] Hamer DH, Chen LH (2014). Chikungunya: Establishing a new home in the western hemisphere. Ann Intern Med.

[15] Lumsden WH (1955). An epidemic of virus disease in Southern Province, Tanganyika Territory, in 1952–53. II. General description and epidemiology. Trans R Soc Trop Med Hyg.

[16] Ross RW (1956). The Newala epidemic. III. The virus’ isolation, pathogenic properties, and relationship to the epidemic. J Hyg.

[17] Silva JVJ, Ludwig-Begall LF, de Oliviera-Filho EF (2018). A scoping review of Chikungunya virus infection, epidemiology, clinical characteristics, viral co-circulation complications, and control. Acta Trop.

[18] Bettis AA, Jackson ML, Yoon IK (2022). The global epidemiology of chikungunya from 1999 to 2020: a systematic literature review to inform the development and introduction of vaccines. PLoS Negl Trop Dis.

[19] Weaver SC, Lecuit M (2015). Chikungunya virus and the global spread of a mosquito-borne disease. N Engl J Med.

[20] Weaver SC (2014). Arrival of chikungunya virus in the New World: prospects for spread and impact on public health. PLoS Negl Trop Dis.

[21] CDC. Areas at risk for chikungunya. 2022. https://www.cdc.gov/chikungunya/geo/index.html. Accessed 26 Oct 2022.

[22] Higgs S, Vanlandingham DL (2015). Chikungunya: here today, where tomorrow?. Int Health.

[23] Rezza G, Nicoletti L, Angelini R (2007). Infection with chikungunya virus in Italy: an outbreak in a temperate region. Lancet.

[24] Chen LH, Wilson ME (2010). Dengue and chikungunya infections in travelers. Curr Opin Infect Dis.

[25] • Rezza G. Chikungunya is back in Italy: 2007–2017. J Travel Med. 2018;25(1). 10.1093/jtm/tay004. **Documents the return of chikungunya infection in Europe and discusses key aspects of importation**. 10.1093/jtm/tay004 29669058

[26] Guzzetta G, Vairo F, Mammone A (2020). Spatial modes for transmission of chikungunya virus during a large chikungunya outbreak in Italy: a modeling analysis. BMC Med.

[27] Pan American Health Organization. Geographic spread of chikungunya in the Americas, December 2013–December 2017. 2018. https://ais.paho.org/phip/viz/ed_chikungunya_amro.asp. Accessed 26 Oct 2022.

[28] Pan American Health Organization. Cases of chikungunya virus disease. 2022. https://www3.paho.org/data/index.php/en/mnu-topics/chikv-en/550-chikv-weekly-en.html. Accessed 26 Oct 2022.

[29] GeoSentinel. The Global Surveillance Network of the ISTM in Partnership with the CDC. 2022. https://www.istm.org/geosentinel. Accessed 26 Oct 2022.

[30] Osman S, Preet R (2020). Dengue, chikungunya and Zika in GeoSentinel surveillance of international travelers: a literature review from 1995 to 2020. J Travel Med.

[31] Kilpatrick AM, Randolph SE (2012). Drivers, dynamics, and control of emerging vector-borne zoonotic diseases. Lancet.

[32] Paixão ES, Teixeira MG, Rodrigues LC (2018). Zika, chikungunya and dengue: the causes and threats of new and emerging arboviral diseases. BMJ Glob Health.

[33] Nasserie T, Brent SE, Tuite AR (2019). Association between air travel and importation of chikungunya into the USA. J Travel Med.

[34] Savini H, Gautret P, Gaudart J (2013). Travel-associated diseases, Indian Ocean islands, 1997–2010. Emerg Infect Dis.

[35] Grobusch MP, Weld L, Goorhuis A (2020). Travel-related infections presenting in Europe: a 20-year analysis of EuroTravNet surveillance data. Lancet Reg Health Eur.

[36] Ammar SE, McIntyre M, Baker MG, Hales S (2021). Imported arboviral infections in New Zealand, 2001 to 2017: a risk factor for local transmission. Travel Med Infect Dis.

[37] CDC (2006). Chikungunya fever diagnosed among international travelers—United States, 2005–2006. MMWR.

[38] CDC (2007). Update: Chikungunya fever diagnosed among international travelers—United States, 2006. MMWR.

[39] Griffiths KM, Savini H, Brouqui P (2018). Surveillance of travel-associated diseases at two referral centres in Marseille, France: a 12-year survey. J Travel Med.

[40] Nakayama E, Tajima S, Kotaki A et al. A summary of the imported cases of Chikungunya fever in Japan from 2006 to June 2016. J Travel Med. 2018;25(1). 10.1093/jtm/tax072. 10.1093/jtm/tax072 29394382

[41] Chou YC, Hsieh CJ, Cheng CA (2020). Epidemiologic characteristics of imported and domestic chikungunya cases in Taiwan: a 13-year retrospective study. Int J Environ Res Public Health.

[42] Norman FF, Henriquez-Camacho C, Diaz-Menendez M, Redivi Study Group (2020). Imported arbovirus infections in Spain, 2009–2018. Emerg Infect Dis.

[43] • Adams LE, Martin SW, Lindsey NP, et al. Epidemiology of dengue, chikungunya, and Zika virus disease in the US states and territories, 2017. Am J Trop Med Hyg. 2019;101(4):884–90. 10.4269/ajtmh.19-0309.** CDC researchers describe 2,284 cases of chikungunya, dengue and Zika, reported in the US and its territories in 2017. Most cases of all three diseases in the US were associated with travel to areas of ongoing virus transmission. The authors call for continuous surveillance to help identify groups at highest risk. **10.4269/ajtmh.19-0309 PMC6779213 31436154

[44] Lindsey NP, Staples JE, Fischer M (2018). Chikungunya virus disease among travelers—United States, 2014–2016. Am J Trop Med Hyg.

[45] •• Staples JE, Hills SL, Powers AM. Travel-related infectious diseases. Chikungunya. In: Centers for Disease Control and Prevention. CDC Yellow Book 2020: Health Information for International Travel. Centers for Disease Control and Prevention. https://wwwnc.cdc.gov/travel/yellowbook/2020/travel-related-infectious-diseases/chikungunya. Accessed 26 Oct 2022. **The CDC’s authoritative handbook for management of travel-related illness, first published in 1967, includes pretravel preparation and destination-specific guidance**.

[46] Gautret P, Mockenhaupt F, Grobusch MP et al. Arboviral and other illnesses in travellers returning from Brazil, June 2013 to May 2016: implications for the 2016 Olympic and Paralympic Games. Euro Surveill. 2016;21(27). 10.2807/1560-7917.ES.2016.21.27.30278. 10.2807/1560-7917.ES.2016.21.27.30278 27416907

[47] Kantele A (2019). Travellers as sentinels of chikungunya epidemics: a family cluster among Finnish travelers to Koh Lanta, Thailand, January 2019. Euro Surveill.

[48] Javelle E, Florescu S, Asgeirsson H (2019). Increased risk of chikungunya infection in travelers to Thailand during ongoing outbreak in tourist areas: cases imported to Europe and the Middle East, early 2019. Euro Surveill.

[49] Diaz-Menéndez M, Esteban ET, Ujiie M (2020). Travel-associated chikungunya acquired in Myanmar in 2019. Euro Surveill.

[50] Dudouet P, Gautret P, Larsen CS (2020). Chikungunya resurgence in the Maldives and risk for importation via tourists to Europe in 2019–2020: a GeoSentinel case series. Trav Med Infect Dis.

[51] • CDC. Chikungunya in the US. 2022. (https://www.cdc.gov/chikungunya/geo/chikungunya-in-the-us.html#:~:text=Chikungunya%20virus%20in%20the%20United,5%E2%80%9265%20per%20year). Accessed 26 Oct 2022. **Summarizes and regularly updates officially reported cases of chikungunya in the USA and its territories, whether travel related or locally acquired**.

[52] CDC Newsroom. First Chikungunya case acquired in the United States reported in Florida. 2014. https://www.cdc.gov/media/releases/2014/p0717-chikungunya.html. Accessed 26 Oct 2022.

[53] CDC (2014). Chikungunya cases identified through passive surveillance and household investigations—Puerto Rico, May 5–August 12, 2014. MMWR.

[54] Feldstein LR, Ellis EM, Rowhani-Rahbar A (2016). The first reported outbreak of chikungunya in the U.S. Virgin Islands, 2014–2015. Am J Trop Med Hyg.

[55] European Centre for Disease Prevention and Control. Annual epidemiologic reports for chikungunya. 2022. https://www.ecdc.europa.eu/en/all-topics-z/chikungunya-virus-disease/surveillance-and-disease-data/annual-epidemiological-reports. Accessed 26 Oct 2022.

[56] CDC (2014). Chikungunya virus spreads in the Americas—Caribbean and South America, 2013–2014. MMWR.

[57] Ali AA, Bajric B, Isache CL, Maharaj RP (2021). Mosquito borne illness in a Floridian hiker. Am J Emerg Med.

[58] Sahadeo N, Mohammed H, Allicock OM (2015). Molecular characterization of chikungunya virus infections in Trinidad and comparison of clinical and laboratory features with dengue and other acute febrile cases. PLoS Negl Trop Dis.

[59] Couderc T, Gangneux N, Chretien F (2012). Chikungunya virus infection of corneal grafts. J Infect Dis.

[60] Petersen LR, Epstein JS (2014). Chikungunya virus—new risk to transfusion safety in the Americas. Transfusion.

[61] Galatas B, Sowath L, Duong V (2016). Long-lasting immune protection and other epidemiological findings after chikungunya emergence in a Cambodian rural community, April 2012. PLoS Negl Trop Dis.

[62] Simon F, Javelle E, Oliver M (2011). Chikungunya virus infection. Curr Infect Dis Rep.

[63] Alvarez MF, Bolívar-Mejía A, Rodriguez-Morales AJ, Ramirez-Vallejo E (2017). Cardiovascular involvement and manifestations of systemic Chikungunya virus infection: a systematic review. F1000Res.

[64] Economopoulou A, Dominguez M, Helynck B (2009). Atypical Chikungunya virus infections: clinical manifestations, mortality and risk factors for severe disease during the 2005–2006 outbreak on Réunion. Epidemiol Infect.

[65] Lemant J, Boisson V, Winer A (2008). Serious acute chikungunya virus infection requiring intensive care during the Réunion Island outbreak in 2005–2006. Crit Care Med.

[66] Traverse E, Hopkins H, Vaidhyanathan V, Barr K (2021). Cardiomyopathy and death following chikungunya infection: an increasingly common outcome. Trop Med Infect Dis.

[67] Cunha MS, Costa PAG, Correa IA (2020). Chikungunya virus: an emergent arbovirus to the South American continent and a continuous threat to the world. Front Microbiol.

[68] Mehta R, Gérardin P, de Brito CAA (2018). The neurological complications of chikungunya virus: a systematic review. Rev Med Virol.

[69] Simon F, Savini H, Parola P (2008). Chikungunya: a paradigm of emergence and globalization of vector-borne diseases. Med Clin North Am.

[70] Cerny T, Schwarz M, Schwarz U (2017). The range of neurological complications in chikungunya fever. Neurocrit Care.

[71] Robin S, Ramful D, Le Seach F (2008). Neurologic manifestations of pediatric chikungunya infection. J Child Neurol.

[72] Gérardin P, Sampériz S, Ramful D (2014). Neurocognitive outcome of children exposed to perinatal mother-to-child Chikungunya virus infection: the CHIMERE cohort study on Réunion Island. PLoS Negl Trop Dis.

[73] Foeller ME, Nosrat C, Krystosik A (2021). Chikungunya infection in pregnancy—reassuring maternal and perinatal outcomes: a retrospective observational study. BJOG.

[74] Duvignaud A, Fianu A, Bertolotti A (2018). Rheumatism and chronic fatigue, the two facets of post-chikungunya disease: the TELECHIK cohort study on Réunion Island. Epidemiol Infect.

[75] Consuegra-Rodriguez MP, Hidalgo-Zambrano DM, Vásquez-Serna H (2018). Post-chikungunya chronic inflammatory rheumatism: follow-up of cases after 1 year of infection in Tolima, Colombia. Travel Med Infect Dis.

[76] van Aalst M, Nelen CM, Goorhuis A (2017). Long-term sequelae of chikungunya virus disease: a systematic review. Trav Med Infect Dis.

[77] Simon F, Watson H, Meynard JB (2021). What chikungunya teaches us about COVID-19. (letter). Lancet Infect Dis.

[78] Paixão ES, Rodrigues LC, Costa MDCN (2018). Chikungunya chronic disease: a systematic review and meta-analysis. Trans R Soc Trop Med Hyg.

[79] Sissoko D, Malvy D, Ezzedine K (2009). Post-epidemic chikungunya disease on Réunion Island: course of rheumatic manifestations and associated factors over a 15-month period. PLoS Negl Trop Dis.

[80] Vidal ERN, Frutuoso LCV, Duarte EC, Peixoto HM (2022). Epidemiological burden of Chikungunya fever in Brazil, 2016 and 2017. Trop Med Int Health.

[81] de Lima STS, de Souza WM, Cavalcante JW (2021). Fatal outcome of chikungunya virus infection in Brazil. Clin Infect Dis.

[82] Vairo F, Haider N, Kock R (2019). Chikungunya: epidemiology, pathogenesis, clinical features, management, and prevention. Infect Dis Clin North Am.

[83] Vicente CR, Cardoso da Silva TC, Pereira LD, Miranda AE (2021). Impact of concurrent epidemics of dengue, chikungunya, zika, and COVID-19. Rev Soc Bras Med Trop.

[84] Furuya-Kanamori L, Liang S, Milinovich G (2016). Co-distribution and co-infection of chikungunya and dengue viruses. BMC Infect Dis.

[85] Prata-Barbosa A, Cleto-Lamane TL, Robaina JR (2018). Co-infection with Zika and Chikungunya viruses associated with fetal death—a case report. Int J Infect Dis.

[86] Mercado-Reyes M, Acosta-Reyes J, Navarro-Lechuga E (2019). Dengue, chikungunya and zika virus co-infection: results of the national surveillance during the zika epidemic in Colombia. Epidemiol Infect.

[87] Eligio-Garcia L, Crisóstomo-Vazquez MDP, Caballero-Garcia MDL (2020). Co-infection of dengue, zika and chikungunya in a group of pregnant women from Tuxtia-Gutierrez, Chiapas: preliminary data, 2019. PLoS Negl Trop Dis.

[88] Schilling S, Emmerich P, Gunther S, Schmidt-Chanasit J (2009). Dengue and chikungunya virus co-infection in a German traveller. J Clin Virol.

[89] Chang SF, Su CL, Shu PY (2010). Concurrent isolation of chikungunya virus and dengue virus from a patient with coinfection resulting from a trip to Singapore. J Clin Microbiol.

[90] Kain D, Jechel DA, Melvin RG (2021). Hematologic parameters of acute dengue fever versus other febrile illnesses in ambulatory returned travelers. Curr Infect Dis Rep.

[91] Natrajan MS, Rojas A, Waggoner JJ (2019). Beyond fever and pain: diagnostic methods for Chikungunya virus. J Clin Microbiol.

[92] Johnson BW, Russell BJ, Goodman CH (2016). Laboratory diagnosis of chikungunya virus infections and commercial sources for diagnostic assays. J Infect Dis.

[93] Musso D, Teissier A, Rouault E (2016). Detection of chikungunya virus in saliva and urine. Virol J.

[94] Martins EB, Silva MFB, Tassinari WS (2022). Detection of chikungunya virus in bodily fluids: the INOVACHIK cohort study. PloS Negl Trop Dis.

[95] Monge P, Vega JM, Sapag AM (2019). Pan-American League of Associations for Rheumatology-Central America, Caribbean and Andean Rheumatology Association consensus—conference endorsements and recommendations on the diagnosis and treatment of chikungunya-related inflammatory arthropathies in Latin America. J Clin Rheumatol.

[96] • Clinicaltrials.gov. Pivotal study to evaluate safety and immunogenicity of a live-attenuated chikungunya virus vaccine candidate in adults. 2022. https://clinicaltrials.gov/ct2/show/NCT04546724. Accessed 26 Oct 2022.. **With phase III trials completed, submission to the Food and Drug Administration for this chikungunya vaccine candidate is expected to be finalized by the end of December 2022**.

[97] Valneva. Valneva successfully completes pivotal phase 3 trial of single-shot chikungunya vaccine candidate. 2022. https://valneva.com/press-release/valneva-successfully-completes-pivotal-phase-3-trial-of-single-shot-chikungunya-vaccine-candidate/. Accessed 26 Oct 2022.

[98] Bennett SR, McCarty JM, Ramanathan R (2022). Safety and immunogenicity of PXVX0317, an aluminum hydroxide-adjuvanted chikungunya virus-like particle vaccine: a randomized, double-blind, parallel group, phase 2 trial. Lancet Infect Dis.

[99] • Clinicaltrials.gov. A phase 3 trial of the VLP-Based chikungunya vaccine PXVX0317. 2022. https://www.clinicaltrials.gov/ct2/show/NCT05072080?cond=chikungunya&draw=2&rank=3VLP. Accessed 26 Oct 2022. **Phase III trials are under way for a virus-like particle chikungunya vaccine candidate with completion expected March 2023 and July 2023**.

[100] Clinicaltrials.gov. Safety and immunogenicity of CHIKV VLP vaccine PXVX0317 in adults ≥65 years. 2022. https://clinicaltrials.gov/ct2/show/NCT05349617. Accessed 26 Oct 2022.

[101] • Clinicaltrials.gov. Phase II study to evaluate safety and immunogenicity of a chikungunya vaccine (MV-CHIK-202). 2021. https://clinicaltrials.gov/ct2/show/NCT02861586. Accessed 26 Oct 2022. **A chikungunya vaccine candidate relies on the same measles-vectored platform used to develop vaccines against SARS and other viruses**.

[102] Reisinger EC, Tschismarov R, Beubler E (2019). Immunogenicity, safety and tolerability of the measles-vectored chikungunya virus vaccine MV-CHK: a double-blind, randomized, placebo-controlled and active-controlled phase 2 trial. Lancet.

[103] Battisti V, Urban E, Langer T (2021). Antivirals against the chikungunya virus. Viruses.

[104] Abdelnabi R, Neyts J, Delang L (2015). Towards antivirals against chikungunya virus. Antiviral Res.

[105] Ferreira AC, Reis PA, de Freitas CS (2019). Beyond members of the Flaviviridae family, sofosbuvir also inhibits chikungunya virus replication. Antimicrob Agents Chemother.

[106] Albulescu IC, White-Scholten L, Tas A (2020). Suramin inhibits chikungunya virus replication by interacting with virions and blocking the early steps of infection. Viruses.

[107] Hucke FIL, Bugert JJ (2020). Current and promising antivirals against chikungunya virus. Front Public Health.

[108] Wichit S, Hamel R, Bernard E (2017). Imipramine inhibits chikungunya virus replication in human skin fibroblasts through interference with intracellular cholesterol trafficking. Sci Rep.

[109] Varghese FS, Kaukinen P, Glasker S (2016). Discovery of berberine, abamectin and ivermectin as antivirals against chikungunya and other alphaviruses. Antiviral Res.

[110] Puntasecca CJ, King CH, LaBeaud AD (2021). Measuring the global burden of chikungunya and Zika viruses: a systematic review. PLoS Negl Trop Dis.

